# A pilot study of yoga as self-care for arthritis in minority communities

**DOI:** 10.1186/1477-7525-11-55

**Published:** 2013-04-02

**Authors:** Kimberly R Middleton, Michael M Ward, Steffany Haaz, Sinthujah Velummylum, Alice Fike, Ana T Acevedo, Gladys Tataw-Ayuketah, Laura Dietz, Barbara B Mittleman, Gwenyth R Wallen

**Affiliations:** 1 National Institutes of Health, Clinical Center, Nursing Department, 10 Center Drive, Bethesda, MD, USA; 2 National Institutes of Health, National Institute of Arthritis and Musculoskeletal and Skin Diseases, 31 Center Dr, Bethesda, MD, USA; 3 National Institutes of Health, Office of Science Policy, Office of the Director, Building 1, Bethesda, MD, USA; 4 National Institutes of Health, Clinical Center, Rehabilitation Medicine, 10 Center Drive, Bethesda, MD, USA; 5 Yoga for Arthritis, Baltimore, MD, USA

**Keywords:** Yoga, Complementary and alternative medicine, Minority, Osteoarthritis, Rheumatoid arthritis, Self-efficacy

## Abstract

**Background:**

While arthritis is the most common cause of disability, non-Hispanic blacks and Hispanics experience worse arthritis impact despite having the same or lower prevalence of arthritis compared to non-Hispanic whites. People with arthritis who exercise regularly have less pain, more energy, and improved sleep, yet arthritis is one of the most common reasons for limiting physical activity. Mind-body interventions, such as yoga, that teach stress management along with physical activity may be well suited for investigation in both osteoarthritis and rheumatoid arthritis. Yoga users are predominantly white, female, and college educated. There are few studies that examine yoga in minority populations; none address arthritis. This paper presents a study protocol examining the feasibility and acceptability of providing yoga to an urban, minority population with arthritis.

**Methods/design:**

In this ongoing pilot study, a convenience sample of 20 minority adults diagnosed with either osteoarthritis or rheumatoid arthritis undergo an 8-week program of yoga classes. It is believed that by attending yoga classes designed for patients with arthritis, with racially concordant instructors; acceptability of yoga as an adjunct to standard arthritis treatment and self-care will be enhanced. Self-care is defined as adopting behaviors that improve physical and mental well-being. This concept is quantified through collecting patient-reported outcome measures related to spiritual growth, health responsibility, interpersonal relations, and stress management. Additional measures collected during this study include: physical function, anxiety/depression, fatigue, sleep disturbance, social roles, and pain; as well as baseline demographic and clinical data. Field notes, quantitative and qualitative data regarding feasibility and acceptability are also collected. Acceptability is determined by response/retention rates, positive qualitative data, and continuing yoga practice after three months.

**Discussion:**

There are a number of challenges in recruiting and retaining participants from a community clinic serving minority populations. Adopting behaviors that improve well-being and quality of life include those that integrate mental health (mind) and physical health (body). Few studies have examined offering integrative modalities to this population. This pilot was undertaken to quantify measures of feasibility and acceptability that will be useful when evaluating future plans for expanding the study of yoga in urban, minority populations with arthritis.

**Trial registration:**

ClinicalTrials.gov: NCT01617421

## Background

In order for an intervention to be used, it must be acceptable to participants, and it must be feasible to administer. Acceptability can be defined as, “that quality which makes an object, person, event, or idea attractive or satisfactory” [1]. Factors highly likely to be influential in shaping values and perceptions include ethnicity, nationality, culture, education, personality, and experience [2]. The purpose of this pilot study is to investigate the feasibility and acceptability of offering a yoga intervention to the urban, minority population served by the National Institute of Arthritis and Musculoskeletal and Skin Disease (NIAMS) Community Health Clinic (CHC) serving the Washington, DC area.

Previous studies using data from the 2002 National Health Interview Survey (NHIS)-Alternative Medicine Supplement found that yoga users were predominately white, female, and college educated with a mean age of 39.5 years [3]. Among those reporting using yoga, 12% were black or Hispanic, 88% stated place of birth as the United States, and 27% used yoga for joint pain [4]. There are a few studies in the literature that examine yoga in minority or diverse populations but none specifically address patients with rheumatic disease [5-7].

This study is a follow-up intervention to a descriptive, exploratory study, completed in 2004 by Wallen et al. (Clinical Trial# http://NCT00069342) which examined the diverse health beliefs and behaviors among a convenience sample of primarily African-American and Hispanic patients enrolled in the NIAMS Natural History of Rheumatic Disease in Minority Communities protocol [8-10]. Overall complementary and alternative medicine (CAM) usage was high, with pain relief listed as the primary reason. A little more than one-third (39%) of respondents stated that they were currently doing movement activity, and only 4.6% stated they were doing yoga [11].

Arthritis is highly prevalent in US adults, with approximately 50 million persons reporting doctor-diagnosed arthritis [12]. Furthermore, arthritis is the nation’s most common cause of disability and is associated with considerable activity limitation, work disability and significant health care costs [13]. The most common form of arthritis is osteoarthritis (OA), a slowly progressive joint disease that occurs when the joint cartilage breaks down. OA symptoms include joint pain, stiffness, knobby swelling, cracking noises with joint movements and decreased function. It typically affects the joints of the hands and spine and weight-bearing joints such as the hips and knees [14,15]. Another form of arthritis is rheumatoid arthritis (RA), an autoimmune, chronic disease that causes pain, stiffness, swelling and limitations in the motion and function of multiple joints. While RA can affect any joint, the small joints in the hands and feet tend be involved more frequently than others [15,16].

Arthritis disproportionally affects certain racial/ethnic minorities. The prevalence of arthritis is lower among blacks and Hispanics than among whites, but the impact is worse. Published analysis of racial/ethnic differences from the NHIS shows the prevalence of activity limitation, work limitation and severe joint pain are significantly higher among blacks, Hispanics, and multi-racial or “other” respondents than among whites [17,18]. People with arthritis should be moving according to the American College of Rheumatology, the Arthritis Foundation and the Centers for Disease Control and Prevention (CDC) [19-21]. People with arthritis who exercise regularly have less pain, more energy, improved sleep and better day-to-day function. Yet, arthritis is one of the most common reasons people give for limiting physical activity and recreational pursuits [20]. People with arthritis may have a difficult time being physically active because of symptoms and lack of confidence in knowing how much and what to do [21]. Long-term studies have shown that people with inflammatory arthritis such as rheumatoid arthritis can benefit from moderate intensity, weight-bearing activity [22]. According the CDC, physical activity can reduce pain and improve function, mobility, mood, and quality of life for most adults with many types of arthritis and recommends including activities that improve balance for people with arthritis who may be at risk for falling [21]. According to the American College of Rheumatology, both range-of-motion (ROM) and stretching exercises help to maintain or improve the flexibility in affected joints and surrounding muscles. This contributes to better posture, reduced risk of injuries and improved function. They recommend activities such as yoga because it incorporates both ROM and stretching movements [22]

According to a report using the data from the 2002 NHIS, adults with arthritis are significantly less likely to engage in recommended levels of physical activity. In both men and women with arthritis, inactivity has been associated with older age, lower education, and having functional limitations. Access to a fitness facility was inversely associated with inactivity. Among women, inactivity was also associated with being Hispanic, non-Hispanic black, having frequent anxiety/depression or social limitations, needing special equipment to increase functional capacity, and not receiving physical activity counseling [23].

Yoga is an ancient system of relaxation, exercise, and healing with origins in Indian philosophy an estimated 5,000 years ago [24,25]. It is regarded as a holistic approach to health reported to increase flexibility, strength, and stamina while also fostering self-awareness and feelings of well-being [26-33]. Yoga can be done anywhere, requires no special equipment, is gentle on the joints and can be modified for each person. It uses only gravity and the body itself as resistance, so it is a low-impact activity. However it is not just an exercise; it is a mind-body intervention. The combination of stress management and gentle physical activity is well suited for investigation in both osteoarthritis (OA) and inflammatory immune-mediated diseases such as rheumatoid arthritis (RA) [34].

Yoga is generally low-impact and safe for healthy people when practiced appropriately under the guidance of a well-trained instructor.  Overall, those who practice yoga have a low rate of side effects, and the risk of serious injury from yoga is quite low [24]. As with any physical activity based on the person undertaking the exercise, there may be contraindications or side effects which make a technique (such as postures or breath work) inadvisable [24,25]. For example poses that put the head lower than the heart may lead to increases in cerebral and intraocular pressure; therefore, patients with high myopia or with hypertension, glaucoma, detachment of the retina, central retinal vein occlusion, discharging ears, or cervical spondylitis should avoid some inverted poses [24,25]. Adverse events and case reports found in the literature were reviewed when preparing the protocol [3,35-37]. However, related poses and techniques reported are not taught or encouraged as part of this protocol. All patients for this study are medically cleared to participate in light to moderate exercise before being recruited to participate.

According to the yogic literature, yoga can help correct the interconnected factors of dysfunctional movement patterns, lack of body awareness, and poor posture [15,26,38]. Yoga takes the whole body through a wide range of motion [15]. Static stretching is the most common technique used in Hatha yoga; which includes both contracting muscles to stretch a target muscle, and when relaxing into a stretch using only body weight to stretch muscles [39]. People with arthritis tend to avoid using sore joints because of the pain involved; inactivity weakens muscles and further decreases range of motion in the joints. Gentle movement taught by a skilled yoga therapist may help by keeping the body moving. Yoga may be suited to help prevent or minimize the erosion of cartilage that causes the joint pain of OA, to create greater ease of movement and decrease pain within joints that have already sustained damage [15]. Most descriptions of the effect of yoga on musculoskeletal disorders point to the benefits of joint realignment and active stretch producing traction of muscles during the asanas (or yoga poses) [40].

A growing number of research studies have shown that the practice of yoga can improve posture, strength, endurance and flexibility [31,41,42]; improve balance, gait, and fear of falling [43-45], and hand grip strength [46]. Other studies have shown that practicing yoga asanas (postures), meditation or a combination of the two, reduces pain for people with arthritis [47,48]. Yoga postures and breath work (pranayama) have been shown to also help with physiological variables such as blood pressure, respiration, and heart rate [47-57]. For patients with arthritis, emphasis on stretching, strength, posture, balance, and the ability to adjust pace and intensity are important components of a safe activity, all of which yoga encompasses. Yoga interventions have been shown to produce improvements in quality-of-life measures related to sense of well-being, energy, and fatigue [58]; and to beneficially impact mood, depression and anxiety disorders [28-33]. Yoga has also been demonstrated to reduce the physical effects of stress [59,60], by reducing the levels of cortisol [61-64]; and affecting the neuroendocrine system [65]. Stress may play a role in worsening symptoms of OA, and contribute to flare-ups of inflammation in RA [15]. There is promising evidence that yoga therapy may help both osteoarthritis and rheumatoid arthritis [25,44,66-75]. The most important limitations of the existing research regarding the impact of yoga are: lack of minority representation, inadequate sample size, overly broad age range, lack of specification regarding the tradition of yoga utilized, and lack of a theoretical model to inform treatment implementation and assessment of outcomes [76].

There are studies that report an interest of diverse minorities in complementary and alternative medicine (CAM) [7] and that CAM is most often used to treat a variety of musculoskeletal problems and conditions [77]. The question to ask is, why not yoga? It is possible that culture plays an important role in the initiation of yoga use and other forms of physical activity. For example, one study found that Latina women’s physical activity depended on their degree of acculturation, expounding that for many the concept of “leisure time,” does not exist. And that middle-class Latinos work five extra hours per week compared with Anglo-Americans [78]. Disparities in the use of yoga in minority groups (specifically low-income populations) could be related to the costs of attending classes. One of the most practiced CAM modalities is prayer, a practice that is financially accessible to all income levels [7,47]. Given that yoga practice can be a fee-based service, it might be challenging for low-income populations to use this health modality. Geographical access to yoga classes may also be problematic for disadvantaged populations. However, a study by Wilson (2008) suggests that given access to yoga practices, diverse populations benefit from these practices, and anticipate using them in everyday life. Almost all participants in this study (98%) would recommend yoga practices to others [7].

The primary objective of this study is to determine the feasibility and acceptability of providing yoga to an urban, minority population with arthritis, using a proven yoga based intervention. The secondary objective is to determine the appropriateness of specific physical and psychosocial measures for this population, and intervention with a focus on physical function and patient reported measures. The tertiary objective is to determine the feasibility of using computerized self-interview (with assistance) to capture baseline and final status.

Assessments will be made from a convenience sample of 20 participants undergoing an 8-week program of yoga classes consisting of 60-minute sessions, twice a week. The yoga classes are designed especially for people with arthritis.

## Methods/design

Research participants will be recruited from English-speaking or Spanish-speaking patients receiving care from the NIAMS rheumatology practice located in Silver Spring, Maryland, a racially diverse area in the Washington DC metro region. Rheumatology care is provided without regard to medical insurance status and patients are referred from other neighborhood health centers, clinics, or practices in the area Potter et al. [79]. Data from the NIAMS Community Health Center (CHC) shows RA (28%) makes up the largest percentage of patients seen followed by OA (11%), and various other rheumatologic diagnoses [80].

Patients must meet all of the following criteria to be eligible for study admission. The Medical Advisory Investigator and nurse practitioner on the study will determine whether subjects meet the medical exclusion criteria:

### Inclusion criteria

• Adult patients enrolled in the NIAMS Natural History of Rheumatic Disease in Minority Communities

• Diagnosis of osteoarthritis (OA) or rheumatoid arthritis (RA)

• Willingness and ability to provide informed consent

• Age ≥ 18 years

### Exclusion Criteria

• Recent (less than 6 months) or planned joint surgery

• Use of assistive ambulatory devices

• Other significant medical or psychiatric conditions, including other inflammatory conditions

• Hyper-mobility or unstable disease that could compromise participation in the study

In attempts to ascertain the initial interest in yoga classes as part of a research protocol, patients from the NIAMS CHC were interviewed in September 2010. Respondents requested to have classes available in both English and Spanish. A common fear expressed about yoga classes was of being required to do “pretzel poses”. Both respondents and NIAMS clinic staff expressed time commitment may be a barrier for this population, due to the need to work several jobs. Most respondents preferred to attend yoga classes from 6–12 weeks.

The study follows 20 participants over an 8-week series of yoga classes. Classes that include deep breathing, relaxation, meditation, poses for strength, flexibility, and balance are offered twice a week. Classes are taught by bilingual (English/Spanish), racially concordant yoga teachers and held at a yoga studio near the NIAMS CHC.

**Figure 1:**
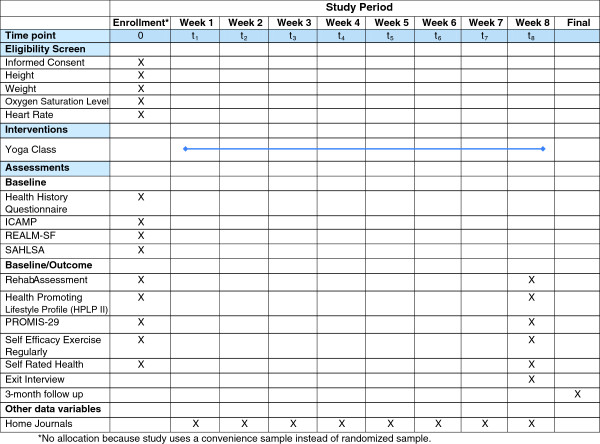
Protocol flow.

At baseline, demographic and clinical data are collected via a computerized in-person interview, conducted by trained interviewers (Figure 1). Patient reported outcomes, captured via a web-based questionnaire, are collected at both baseline and the end of the study. Physical measurements, also collected at baseline and the end of the study, are obtained by rehabilitation medicine. Field notes, quantitative and qualitative data regarding feasibility and acceptability are also collected during the course of the study.

For this pilot intervention, class size is kept small (5–10 participants) to allow for modifications and explanation of yoga poses that will encourage greater self and body awareness. The yoga therapy approach tailors each pose to the needs and limitations of every individual. Props (chairs, bolsters, blankets, blocks and straps) are used and the postures are modified to accommodate the limitations of the arthritis patients taking the yoga classes.

Participants are encouraged to develop a home practice, which appears to be critical to the effectiveness of the intervention [15]. The last section of each class is dedicated to providing guidance on how to do home practice based on poses taught and information given during each yoga class. All participants receive a yoga mat, blocks, belt, and blanket for their home practice. Participants keep a journal to qualitatively document the frequency of home practice and their experience of participating in the study. Journals are open format, allowing respondents to record their observations/feelings about practicing yoga in their own words [81-84].

This study focuses on the branch of Hatha yoga that uses postures (asanas), breathing techniques (pranayama) and meditation. Within Hatha yoga, there are numerous styles each with a slightly different approach to the physical practice of yoga. This protocol is primarily influenced by the styles of Integral, Iyengar and Kripalu yoga, each of which is considered to focus on proper physical alignment in a gentle and attentive fashion, while also including a strong component of mindfulness during and between physical postures [15,85].

The yoga classes are formatted so that every class builds on the previous class. The contents of class 1 are repeated with additional poses added during each successive class (Table 1).

**Table 1:** Overview of yoga poses

	**Description**	**Yoga poses**
**Laying foundation:**		
**Classes 1-2**	Warm-up:	Upper body stretches, staff with leg lifts
	Sun Salutations (one side):	Forward fold, mountain (two sides for class 2)
	Standing poses:	Tree, warrior II
	Sitting poses:	Head to knee, spinal twist, yogic seal
	Relaxation:	Sivasana, tense and release, progressive body scan
	Closing:	Side lying, cross-legged
**Class 3**	Discussion of balance poses	Tree, king dancer
**Classes 4-5**	Arm balancing and reclining poses	Inverted plank, (lying) extended leg pose, (lying) spinal twist
**Classes 6-7**	Arm/leg extensions and hip openers	Table and cat/cow-extend arm & opposite leg, downward facing dog-extending one leg, bridge with leg extension, butterfly
**Classes 8-9**	Intro to gentle back bends	Sphinx, locust, bow, camel
**Classes 9-10**	Stamina building	Four sun salutations
**Class 11**		Poses for sciatica
**Class 12**		Pose modifications using the wall
**Class 13**		Restorative poses
**Classes 14-16**		Review, practice, wrap up

This format follows that of the previous randomized research study *Yoga for Arthritis*, conducted through Johns Hopkins University, which explored mediators of yoga practice and health related quality of life measures and disease symptoms in RA and OA patients. Results from the study found benefits of this sixteen-class yoga series created specifically for those with arthritis included:

• A statistically significant improvement in overall physical health, flexibility, and balance.

• A significant reduction in symptoms of depression and improvement in positive affect.

• A significant improvement in pain symptoms and, for those with RA, a significant difference in the number of tender and swollen joints when compared with control subjects receiving the usual medical care [86,87].

Dr. Steffany Haaz, a researcher on the Hopkins study and an advanced yoga teacher trained in yoga therapy provided yoga for arthritis training to bilingual yoga instructors recruited to teach for this study. In order to optimize treatment fidelity, all instructors use the same training manual of 16-class sequence used in the Haaz study and all classes are videotaped. Class attendance, the name of yoga instructor, home practice, and reported side effects are recorded during each class session.

The primary aim of this pilot study is to attempt to quantify measures of feasibility and acceptability that will be useful when evaluating future plans for expanding the study of yoga in this population. There are a number of challenges in recruiting and retaining study participants in a community setting. We hypothesize that by providing bilingual materials, racially concordant images for recruitment materials, culturally similar investigators and yoga instructors, as well as an intervention created specifically for persons with arthritis; participants will be more willing to enroll, attend classes, and report a positive experience after completing the 8-week series.

The second aim of this study is to determine if the measures of well-being selected for this minority population are appropriate to discern facilitators and impediments for participants to view yoga as a viable option for self-care. Self-care has become increasingly important in being able to self-manage chronic disease states; however, many patients with chronic disease do not integrate self-management behaviors into their lives [88]. The concept of well-being integrates mental health (mind) and physical health (body) resulting in more holistic approaches to disease prevention and health promotion [89]. Well-being includes the presence of positive emotions and moods (e.g., contentment, happiness), and the absence of negative emotions (e.g., depression, anxiety), as well as aspects of physical, social, emotional, psychological well-being [89]. It is associated with: self-perceived health, healthy behaviors, mental and physical illness, and social connectedness [89]. For this study, self-care is defined as adopting behaviors (such as continuing to practice yoga) that improve physical and mental well-being and may decrease arthritis symptoms and side effects.

**Figure 2:**
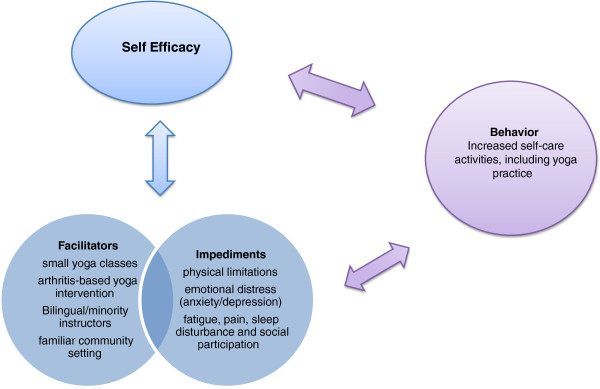
Self care model.

A revised model based on Bandura’s social cognitive theory (Figure 2) shows the paths of influence believed to be relevant for this study. Bandura’s (2004) theory suggests that self-efficacy plays an important role in motivating behavior change [90]. Self-efficacy is the confidence in one’s personal ability to perform a task or behavior or to change a specific cognitive state, regardless of circumstances or contexts [91,92]. Social cognitive theory specifies a core set of determinants, among which perceived self-efficacy and knowledge of p*erceived facilitators* and *impediments* may lead to behavior changes [90,93]. Ways to influence self-efficacy include learning a new behavior, seeing people similar to oneself succeed; social persuasion; reducing negative emotional states; and correcting misinterpretations of physical ability [90,93]. The potential facilitators and impediments are measured at baseline and again at the end of the study to see if there are any changes after completing the intervention.

The third aim is to determine the feasibility of using computerized self-interview (with assistance) to capture baseline and final health status outcomes. The previous study in this population by Wallen was completed using an in-person paper-and-pencil interview questionnaire [9,10]. In attempts to determine the feasibility for using a computerized system, this study uses a computer-assisted interview method with both the interviewer collecting baseline background information; and the respondent entering patient reported outcome measures directly into a web-based system, with assistance as needed.

Acceptability of this study will be evaluated based on the response rate, percent of classes completed, exit interview comments and the percent of patients continuing yoga after three months. Feasibility will be determined based on exit interview (see Appendix) comments and qualitative data. Qualitative field notes are kept to monitor/document related issues such as location, personnel, equipment, and the amount of modifications needed during yoga classes. Records are kept of eligible patients who decline and reasons for declining. Field notes are maintained by study investigators during recruitment, onsite yoga classes, and when interacting with study participants outside of completing questionnaires. An exit interview is completed after the last class. Those who may have dropped out of the study prior to the last class will be contacted by phone in attempts to complete the exit interview.

The feasibility of using a computerized self-interview method is based on the estimated amount of assistance needed to complete questionnaires (measured in increments from 0-100%), comments collected from the interviewer offering assistance, any technical problems encountered, and comments solicited from the participants during the exit interview.

#### Baseline assessment

Self-reported demographic variables include: gender, age, marital status, race/ethnicity, educational level and occupational status. Clinical variables related to arthritis and current practices include: rheumatic diagnosis, other diagnoses, weight, height, duration (years) of rheumatic disease and location current joint stiffness and pain location [94], current medications, heart rate and oxygen saturation level, exercise, diet and an Inventory of Complementary and Alternative Medicine Practices (ICAMP) created during the Wallen study [9]. Acculturation is measured by obtaining the proxy measures of spoken English language proficiency, country of origin, and length of time in United States.

*Health literacy* is the degree to which individuals have the capacity to obtain, process, and understand basic health information and services needed to make appropriate health decisions. This study uses the Rapid Estimate of Adult Literacy in Medicine Short Form (REALM-SF) and Short Assessment of Health Literacy for Spanish Adults (SAHLSA-50) tools [95-98]. The REALM is an assessment tool using word recognition tests, which has high criterion validity when correlated with other literacy tests and has high test-retest and reliability 0.97 (p < .001) [96-98]. The REALM-SF is a 7-item word recognition test with scores that were highly correlated with the REALM in development (r = 0.95, p < 0.001) and validation (r = 0.94, p < 0.001) samples [99]. The SAHLSA-50 is a validated health literacy assessment tool containing 50 items designed to assess a Spanish-speaking adult’s ability to read and understand common medical terms, based on REALM [100]. The SAHLSA-50 correlated with the Test of Functional Health Literacy in Adults(r = 0.65); and displayed good internal reliability (Cronbach’s alpha = 0.92) and test-retest reliability (Pearson’s r = 0.86) [101].

### Patient reported outcomes and psychosocial concepts

Patient reported outcomes are assessed at baseline and at the end of the 8-week yoga intervention, with bilingual staff available to help complete documentation and computer usage. In attempts to reduce participant burden, instead of using legacy measures, this study is using the Patient-Reported Outcomes Measurement System (PROMIS). The item databanks for PROMIS have been tested for reliability and comparability using Item-Response Theory; short forms were developed and compared with other well-validated and widely accepted (“legacy”) measures [102-104]. The PROMIS 29-profile was selected as a domain framework for self-reported health to look at eight domains: physical function, anxiety, depression, fatigue, sleep disturbance, satisfaction with social roles, pain interference, and pain intensity.

S*elf-care and well-being* are evaluated using the Health-Promoting Lifestyle Profile II (HPLP-II), self-efficacy, self-rated health, class attendance, reports of continued practice yoga after completing the study intervention, as well as qualitative data (participant comments). The HPLP-II is a self-administered 52-item instrument that measures the frequency of self-reported healthy behaviors. It consists of 6 subscales: physical activity, spiritual growth, health responsibility, interpersonal relations, nutrition, and stress management. This is a 4-point Likert type scale with responses ranging from 1 (never) to 4 (routinely). The higher scores indicate the more frequent engagement in health behaviors [105]. Callaghan (2003) reported the following Cronbach’s alpha coefficients of internal consistency reliability: total scale 0.93, health responsibility 0.83, physical activity 0.87, nutrition 0.76, spiritual growth 0.84, interpersonal relations 0.82, and stress management 0.75 [106]. A randomized study of bilingual Hispanic individuals found the English- and Spanish-language versions of the HPLP II to have statistically acceptable levels of reliability and equivalency [107].

*Self-efficacy* is measured using Lorig’s self-efficacy in exercise, a 3-item scale used to measure confidence in exercising regularly based on a scale from 1 (not at all confident) to 10 (totally confident) [108]. Higher scores indicate more confidence. Lorig et al. [109] and Wallen [9], have validated the 8-item Arthritis Self-Efficacy Scale (ASES) with similar populations, however the Haaz Y*oga for Arthritis* study [110] found no change when using the same ASES measure. For this pilot, the decision was made to focus specifically on using a measure related to exercise self-efficacy.

*Self-rated health* consists of a single response item, “Would you say your health in general is excellent, very good, good, fair, or poor?” [108]. Lorig et al. (1996) found the mean rating for this item to be 3.29 (*SD* ± 0.91). In a subset of their sample (*n* = 51), this item displayed a test-retest reliability of .92 [108,111]. This measure has been translated into Spanish [111]. Self-rated/self-reported health (SRH) has been differentially reported by Hispanics compared to whites, especially based on their acculturation status [111-114]. Hispanics are 3.6 times more likely to report fair or poor health compared to whites [115].

*Physical assessment* -Clinical measures were selected to evaluate domains of balance (single leg stance, functional reach test) and functional mobility (timed up and go test) that may be responsive to change with an exercise intervention. These measures are evaluated both at baseline and after completing the series of yoga classes, by the National Institutes of Health (NIH) rehabilitation medicine staff associated with the study. The *single leg stance* (SLS) determines if the patient can stand on one leg for 10 seconds [116]. When evaluated mean criterion-related validity was high (Pearson’s r =0.84, 0.83, respectively). Inter-observer reliability was high (ICC (2,1) = 0.81 at Test 1 and 0.82 at Test 2). Intra-observer reliability was high (on average ICC (2,1) = 0.88; Pearson’s r = 0.90) [117].

The *functional reach test* is a dynamic measure of stability during a self-initiated movement [118]. Functional reach is the difference (in inches) between a person’s arm length and maximal forward reach with the shoulder flexed to 90 degrees while maintaining a fixed base of support in standing. In a study by Duncan (1990), it was found that functional reach measures were strongly associated with measurements of the center of pressure excursion. The Pearson correlation coefficient was 0.71 and the *R2* using linear regression was 0.51. Analysis of the test-retest reliability of the three primary measures of postural control suggests that functional reach is highly reproducible. The intraclass correlation coefficient (ICC 1, 3) for center of pressure excursion was 0.52, electronic functional reach 0.81, and “yardstick” reach 0.92. [119].

The *timed “Up and Go” test* (TUG) measures, in seconds, the time taken by an individual to stand up from a standard arm chair, walk a distance of 3 meters, turn, walk back to the chair, and sit down [120]. A history of arthritis increases the risk of falling, sensitivity of the TUG for predicting falls was 0.80 and specificity was 0.934 [121,122]. Inter-rater reliability for the TUG is high with a same day, three-rater intra-class correlation coefficient (ICC) of various studies has ranged from 0.99 - 0.992. For validity, moderate to high correlations have been observed with scores on Berg Balance Scale, gait speed, stair climbing, and the Barthel Index of Activities of Daily Living Scale [121].

A *timed floor transfer* is a clinical test of strength, flexibility, function, and problem solving; it measures the time necessary to transfer from standing to the floor and return to standing in any way that participants are able [123]. The time needed is recorded in seconds, then standardized by using body height to determine the speed of this task. Higher values indicate better scores [124]. The interrater reliability for three timed tests (50-ft walk, five-step, and floor transfer) between various pairs of two testers was determined to be excellent (r = .99) [124]. The calculated ICCs were moderately high (ICC_2,1_ = .79, *p* = .0001) on the timed data to determine test–retest reliability in a community-based, predominantly Hispanic population some of whom were diagnosed with arthritis [125].

U*pper body ability* is measured using the Disabilities of the Arm, Shoulder and Hand (DASH) Outcome Measure. The DASH is a 30 item self-report questionnaire designed to measure physical function and symptoms in patients with any or several musculoskeletal disorders of the upper limb. The DASH has been shown to have acceptable reliability and validity when assessing upper limb functional ability in RA populations [126]. A study undertaken to evaluate the reliability, validity, and responsiveness of the DASH found it to correlate with other measures (r > 0.69) with a test-retest reliability of (ICC = 0.96) [127].

The study is a pilot convenience sample of 20 participants. Yoga classes will be held for as few as 5, or as many as 10 participants per class depending on how long it takes to accrue participants onto the study. Research participants are recruited from English-speaking or Spanish-speaking patients receiving care from the NIAMS CHC rheumatology practice.

Racially concordant images were used to develop pamphlets to be displayed in clinic and used for recruitment. All recruitment materials are available in both English and Spanish due to the large percentage (57%) of Latino/Hispanic patients seen by the NIAMS CHC (personal communication: Alice Fike MSN). Because of clinician’s concern regarding literacy, all documents are read to patients in either English or Spanish, when needed. Patients are referred to the Principal Investigator by the NIAMS rheumatology clinicians who are already familiar with their care. After full study explanation, either the Principal Investigator or Lead Associate Investigator obtain written informed consent in person at the rheumatology clinic.

Participant information will be collected using a computerized questionnaire during one-on-one sessions with either the Principal Investigator or Associate Investigators. Data will be collected electronically using the Clinical Trials Database (CTDB). CTDB is a web based application that is hosted at the NIH therefore data are housed on a dedicated in-house server protected according to federal standards. The security framework of CTDB and the clinical trials survey system (CTSS) permit only the Principal Investigator and designated Associate Investigators to have access to identified data in the database.

This pilot study evaluates the acceptability of offering yoga as an integrative intervention for self-care using the response rate, percent of classes completed, exit interview comments and the percent of patients continuing yoga after three months. Barriers and reasons for continuing/discontinuing yoga are assessed in a brief questionnaire either in person or by telephone, three (3) months after completing yoga classes. Results from the Haaz study were used to determine reasonable measure and time frames for this study. The study found of 102 patients screened, 52% of randomized persons completed the 8-week yoga session [128]. Those who actually started the intervention were very likely to complete it, as attrition was highest prior to the first class [129]. Based on these results, the decision was made to decrease the time from enrollment to start of yoga classes; and to decrease the class size to 5–10 participants per class.

Acceptability of this study will be evaluated based on the response rate, percent of classes completed, exit interview comments and the percent of patients continuing yoga after 3 months. Feasibility will be determined based on exit interview comments and qualitative data. Qualitative field notes will be kept to monitor/document related to issues such as site capability (location/space), personnel (bilingual yoga teachers/investigators), equipment (computers/yoga props), and the amount of modifications needed. A record will be kept of eligible patients who decline and reasons for declining.

Statistical Package for the Social Sciences (SPSS) Version 20.0 will be used to analyze outcome measure data. Descriptive statistics (mean and median) will be used to describe the profiles of the subjects from demographic data gathered. Wilcoxon tests will be used to test differences between baseline and final assessments, for those who complete the study. Pre- and post- changes will be identified; however, no attempt will be made to evaluate statistical significance given the small sample size of the pilot study. An attempt will be made to complete an exit interview for all participants, even those who discontinue participation in the study prior to full completion. Missing data will be managed according to the recommendations of selected measures used in the protocol, or omitted from analyses.

The feasibility of using a computerized self-interview method (with assistance) will be based on the percent of assistance needed to complete questionnaires, comments collected from the interviewer offering assistance and comments solicited from the participants during the exit interview.

The Medical Advisory Investigator (MAI) will be responsible for the patient safety monitoring. The Principal Investigator will provide a regular report of all protocol activities to the MAI and discuss any concerns. No invasive treatments are provided as part of this protocol. This population will not be exposed to yoga poses (i.e. headstands and shoulder stands) that are most commonly associated with injury. Adverse events and serious adverse events will be monitored at all times during yoga classes and if reported by subjects by telephone. Specific safety instructions will be given as part of every class and pose modifications will be taught to individuals as needed. Participants will be instructed to report side effects immediately should they occur.

Investigators will attend all yoga classes and will ask subjects about side effects during each class session. Subjects will also be instructed to report side effects immediately, to the study PI; by telephone should they occur while practicing at home. Any reported side effects will be documented by the investigators using the weekly flow sheet and reviewed daily by the PI. The PI will report adverse events to the MAI for recommendations and follow-up, and record in the patient record. For research related injury, the subject will be evaluated by NIAMS rheumatology at either the NIAMS CHC or the NIH Clinical Center.

A serious adverse event is defined as any untoward medical occurrences that 1) result in death, 2) are life-threatening, 3) require hospitalization, 4) cause persistent or significant disability/incapacity, 5) result in congenital anomalies or birth defects, 6) are other conditions which in the judgment of the Investigators represent significant hazards. All serious adverse events will be reported to the IRB and to the Clinical Director, within 7 days for death or life threatening adverse event and within 15 days for all others. All adverse events reported under this protocol will be limited to those events which are possibly, probably or definitely related to the research described in this protocol.

Under the Natural History protocol (Clinical Trial# http://NCT00024479), the reporting of adverse events will focus on adverse events that are related to the diagnostic and therapeutic interventions where the NIH is involved either directly or indirectly by recommending certain interventions. Adverse events associated with the natural history of rheumatic disease will not be reported. Adverse events associated with the yoga intervention will be monitored by the PI, serving as safety officer, and will be reported to the IRB as appropriate and as part of the annual report/renewal.

Approval to conduct the study was obtained through the National Institute of Diabetes and Digestive and Kidney Disease/National Institute of Arthritis and Musculoskeletal and Skin Disease’s intramural Institutional Review Board. Since the protocol approval, an amendment was submitted to include a Spanish consent, to obtain approval for a change in the location for offering the yoga classes and to add a yoga student manual for participants on the study. This amendment was approved on 6/4/2012. A second amendment was submitted during the continuing review process to remove one associate investigator and add three new investigators. This amendment was approved on approved 2/20/2013.

Consent is obtained by the Principal Investigator or Lead Associate Investigator. Once the study has been explained to subjects, including the objectives, time commitment and process, subjects are given the informed consent/assent document to review. Subjects are encouraged to ask questions prior to enrolling in this study. Subjects are reassured that participation in this study is entirely voluntary and that they may withdraw from the study at any time. Subjects are informed that their decision to participate in or withdraw from this study will impact neither their participation in other protocols for which they may be eligible, nor their ability to receive services at the Clinical Center that they may require.

Patient data including the results of physical function tests and responses to questionnaires are entered into an NIH-authorized and controlled research database. Any future research use will occur only after appropriate human subject protection institutional approval as prospective NIH IRB review and approval an exemption from the NIH Office of Human Subjects Research Protections. The Principal Investigator is responsible for overseeing entry of data into an in-house password protected electronic system and ensuring data accuracy, consistency and timeliness. The Principal Investigator, Associate Investigators and/or a contracted data manager will assist with the data management efforts.

All human subjects’ personally identifiable information as defined in accordance to the Health Insurance Portability and Accountability Act (HIPAA) will be separated from individual subject data. Protocol eligibility and consent verification will be tracked and separated from individual subject data. Primary data obtained during the conduct of the protocol will be kept in secure network drives that comply with NIH security standards. Primary and final analyzed data will have identifiers so that research data can be attributed to an individual human subject participant required for subject identification, e.g., study-specific identifying number generated by Principal Investigator and/or Associate Investigators for subject identification. The protocol and all primary and analyzed data will be stored in the NIH Clinical Center’s secure network*.* Clinical data will be collected using subjects’ names in the source document. However, clinical report forms will be coded. Research survey responses, will be maintained in a secure network password-protected database (Clinical Trials Database (CTDB)). Any printed records with identifier information will be kept in a locked file cabinet within a secure file cabinet of the PI.

Investigators will be responsible for collecting the questionnaires from subjects and ensuring the delivery of the data to the secure office of the Principal Investigator. Data from consenting subjects will be stored until they are no longer of scientific value or if a subject withdraws consent for their continued use, at which time they will be destroyed. Should we become aware that a major breach in our plan for tracking and storage of data has occurred, the IRB will be notified. Each NIH protocol undergoes a yearly departmental independent audit in addition to yearly continuing reviews by the IRB.

Since this protocol is federally funded, any manuscript to be published in a peer-reviewed journal will be submitted to PubMedCentral or public access upon acceptance for publication.

## Discussion

There are a number of challenges in recruiting and retaining participants from a community clinic serving minority populations. Few studies have examined offering integrative modalities to this population. This pilot was undertaken in order to quantify measures of feasibility and acceptability that will be useful when evaluating future plans for expanding the study of yoga in an urban, minority population with arthritis.

Previous studies of patients with arthritis suggest that taking yoga classes helps to improve measures of physical health, flexibility, balance, affect, and pain symptoms as well as reduces measures of depression. It is unclear what aspects of yoga as an intervention may or may not be acceptable to populations such as those served by the NIAMS CHC.

Knowledge gained from this pilot study will contribute to the understanding of the feasibility of this study design and the acceptability of yoga as self-care modality for minorities with arthritis. The design of the study will also add to the body of work related to the use of patient reported outcome measures and the use of computerized data collection methods within this population.

## Appendix: yoga study exit interview

Thank you for participating in our study. We would like to ask you some questions as you are finishing the study. Please give your honest answers to help us improve our study. Your comments are very important to us.

1. We would like to ask about your experience using the laptop computer to complete the surveys throughout the study.

a. How comfortable were you using the laptop computer? *Please circle the appropriate number below*:

Very Comfortable 1 2 3 4 5 Very Uncomfortable Comfortable

b. Do you think we should continue use computers in this way?

○ Yes

○ No

c. Is there anything else you would like us to know about your experience using the computer to complete the surveys in this study?

2. Please give us your opinion on the yoga classes and location:

a. Overall, how satisfied were you with the yoga classes? *Check one:*

○ Completely satisfied

○ Mostly satisfied

○ Equally satisfied and dissatisfied

○ Mostly dissatisfied

○ Completely dissatisfied

○ Unsure

b. How satisfied were you with the classroom where the classes were offered? *Check one:*

○ Completely satisfied

○ Mostly satisfied

○ Equally satisfied and dissatisfied

○ Mostly dissatisfied

○ Completely dissatisfied

○ Unsure

3. How much do you agree or disagree with each of the following statements? *Please circle one of the choices below.*

(SA).....Strongly agree

(A).......Agree

(N).......Neither agree nor disagree

(D).......Disagree

(SD).....Strongly Disagree

a. Yoga classes should be offered in English only

SA.….....A…......N………D….…..SD

b. Yoga classes should be offered in both English and Spanish

SA.….....A…......N………D….…..SD

c. I feel more comfortable taking yoga classes from teachers with diverse racial/ethnic backgrounds

SA.….....A…......N………D….…..SD

d. prefer taking yoga classes with others who have arthritis

SA.….....A…......N………D….…..SD

e. The yoga poses offered in class work well for people with arthritis

SA.….....A…......N………D….…..SD

4. What did you like most about the yoga classes?

5. What did you like least about the yoga classes?

6. What changes would you make in the classes to make them more helpful to your arthritis?

7. Would you recommend yoga classes to a friend with arthritis?

○ Yes

○ No

8. Now that you have completed the study, do you see yoga as a way to care for your arthritis? *Please check one of the circles below and answer the following questions*.

○ Yes –please tell us why you see yoga as a way to care for your arthritis.

○ No – please tell us why you do not see yoga as a way to care for your arthritis.

9. How likely do you think it is that you would take another yoga class? *Check one:*

○ Extremely likely

○ Fairly likely

○ Somewhat likely

○ Slightly likely

○ Not at all*

○ Unsure

*If you answered “not at all” above, please tell us why (*for example: cost too much, too far away, don’t have time, no longer interested in yoga*).

10. How likely do you think it is that you would practice yoga on your own now? *Check one:*

○ Extremely likely

○ Fairly likely

○ Somewhat likely

○ Slightly likely

○ Not at all*

○ Unsure

*If you answered “not at all” above, please tell us why (*for example: do not have time, no longer interested in yoga*).

## Abbreviations

ASES: Arthritis self-efficacy scale; CAM: Complementary and alternative medicine; CDC: Centers For Disease Control And Prevention; CHC: Community health center; DASH: Disabilities of the arm, shoulder and hand; HPLP-II: Health-Promoting Lifestyle Profile II; ICAMP: Inventory of complementary and alternative medicine practices; ICC: Intraclass correlation coefficients; NIH: National Institutes Of Health; NHIS: National Health Interview Survey; NIAMS: National institute of arthritis and musculoskeletal and skin diseases; OA: Osteoarthritis; PROMIS: Patient-Reported Outcomes Measurement System; RA: Rheumatoid arthritis; REALM-SF: Rapid estimate of adult literacy in medicine - short form; SAHLSA-50: Short assessment of health literacy for Spanish adults −50; ROM: Range-of-motion; SLS: Single leg stance; SRH: Self rated health; TUG: Timed *“Up and Go” test.*

## Competing interests

The authors declare that they have no competing interests.

## Authors’ contribution

KM and GW and are accountable for data acquisition and preparation of the manuscript. KM, GW and MW designed the study. All authors contributed to the writing and preparation of the study protocol, excerpts of which were used in creating this manuscript. All authors have read and approved the final manuscript.
